# Indian Association of Occupational Health denies industry influence

**DOI:** 10.4103/0019-5278.43268

**Published:** 2008-08

**Authors:** S. M. Shanbhag

**Affiliations:** President, Indian Association of Occupational Health, L B S Marg, Mulund West, Mumbai-400 080, India. E-mail: shrinivas.shanbhag@ril.com

From : Editor's desk

Readers may be aware of Dr. T.K. Joshi's letter published in Int. J. of Occup Environ Health, 2008; 14;82, titled “Industry influence on IAOH.” Dr. S.M. Shanbhag, National President of IAOH had responded to the Editor of the Int. J. of Occup Environ Health (Published in Int. J. of Occup Environ Health, April/June2008; Vol 14/No2,pg159). We reproduce the IAOH President's response for the benefit of our readers

Dear Sir,

We write in response to Dr. Tushar Kant Joshi's letter on the “Industry Influence on Indian Association of Occupational Health”.[1] The Indian Association of Occupational Health (IAOH) is the largest non-governmental organization working in the field of occupational health in India. The association is more than 60 years old has a membership of more than 2800 spread over 24 branches across the country.

The mission statement of IAOH is “To be a nodal non governmental organization committed to attain highest standards of Occupational Health in India by enabling stakeholders, influencing policy makers and creating community consciousness.” The association is committed to further the cause of occupational health in the country and does so by means of scientific meetings, conferences, and through trying to influence policy decisions in the area of occupational and environmental health. The association also encourages its members to present scientific papers and best practices during the conferences. The IAOH enjoys consultative status with the World Health Organization.

The issues raised by Dr. Joshi are baseless and blatantly false, and the IAOH denies them. Dr. Joshi, while writing the letter must surely have been driven by motivated self interest and has hence chosen to tarnish the image of the IAOH by leveling malicious allegations. I do not understand how Dr. Joshi has chosen to accept awards and orations from the IAOH even though he holds such a poor opinion of the association. The association has enough communications from Dr. Joshi on record, where he has chosen to compliment the association for supporting him. To us his actions are akin to the pot calling the kettle black!

IAOH is a not for profit organization and has to depend on sponsorships and donations like all NGOs for carrying out the activities to achieve its objectives. (One wonders who funds the Ban Asbestos movement and under whose influence this lobby works when individuals like Dr. Joshi talk about occupational health practice with integrity!) The IAOH takes utmost care to ensure that there is no quid pro quo whilst accepting such sponsorships. The association does not accept sponsorships from corporations who manufacture harmful products like tobacco. After 2003, when the Central Council decided to support the Ban Asbestos movement based on scientific evidence, the association has also directed all its branches hosting conferences not to accept sponsorships from companies dealing with asbestos.

The official organ of the association, the Indian Journal of Occupational and Environmental Medicine, has also published an editorial supporting the banning of asbestos.[2] The journal had also invited Dr. Joshi to write an editorial entitled “Precautionary Principle and need to ban all forms of asbestos use in India,”[3] which was published in 2002.

As a senior member of the Association and the Past President of our Delhi Branch, Dr. Joshi is well aware of the above facts. I therefore wonder what drove him to write the said letter. I am also surprised that a reputable journal like IJOEH decided to print the letter from Dr. Joshi without seeking any response from IAOH. The Association strongly believes that scientific deliberations are the right way forward and does not believe in sensationalizing or politicizing any occupational health issue.
